# COVID-19 vaccine allocation: addressing the United Kingdom’s colour-blind strategy

**DOI:** 10.1177/01410768211001581

**Published:** 2021-03-09

**Authors:** Tasnime Osama, Mohammad S Razai, Azeem Majeed

**Affiliations:** 1 Department of Primary Care & Public Health, School of Public Health, 4615 Imperial College London, London W6 8RP, UK; 2 Population Health Research Institute, St George’s University of London, London SW17 0RE, UK

COVID-19 has disproportionately affected Black, Asian and Minority Ethnic (BAME) groups,^1^ resulting in higher rates of infection, hospitalisation and death.^1^ The pandemic has also exposed the pre-existing racial and socioeconomic inequalities in the UK.^2^ However, the Joint Committee on Vaccination and Immunisation has omitted ethnic minorities from the top priority groups which include older age, frontline health and social care workers, and care home staff and residents.^3^ The invisibility of these vulnerable groups from the priority list and the worsening healthcare inequities and inequalities are putting ethnic minorities at a significantly higher risk of COVID-19 illness and death.

The UK’s colour-blind vaccination model disregards the unequal impact of the pandemic on minority ethnic groups, rendering it an enabler of structures that are known to systematically disadvantage BAME communities. Furthermore, the UK currently has one of the highest death rates from COVID-19 globally.^4^ With the new UK variant, B.1.1.7, being 70% more transmissible and 30% more deadly,^5^ the virus will continue to have a larger detrimental impact on BAME groups. It is, therefore, essential that the UK grounds its priority-related decisions in real-time evidence to ensure that ethical vaccine allocation and local barriers to vaccination uptake by BAME communities are addressed and prioritised nationally, thereby preventing further exacerbation of inequalities during the pandemic and in its aftermath.

## The case against colour-blind vaccine allocation strategies: uneven starting points in BAME groups

The racial disparity in COVID-19 first became apparent in the UK when the first 11 doctors to die from COVID-19 were all of BAME background.^6^ Subsequent global real-time evidence continued to demonstrate that BAME groups have higher rates of COVID-19 infection, admission to the intensive care unit and death.^1^ After adjusting for age, for example, Black men and women are 4.2 and 4.3 times as likely to die from COVID-19 when compared to their White counterparts, respectively.^7^ Similarly, Bangladeshi and Pakistani men and women are 3.6 and 3.4 times as likely to die from COVID-19, respectively.^7^ The relationship between ethnicity and COVID-19-related clinical outcomes appear to be largely explained by the social determinants of health, including systemic racism and socioeconomic differences, rather than genetics or biology; BAME communities are worse-off due to epidemiological, economic and social issues. If insufficient numbers of individuals from BAME communities are vaccinated, the virus will continue to spread among these groups, further increasing the health divide and, ultimately, putting the general population at risk.

People from minority ethnic groups are more likely to live in crowded and multi-generational households where self-isolation and social distancing may prove to be difficult, or even impossible, thereby increasing transmission risk.^8^ Individuals living in deprived areas have higher diagnosis and death rates, with the mortality rate of those living in the most deprived areas being more than double that of those living in the least deprived areas of England.^9^ Previous evidence from the 2009 H1N1 swine flu pandemic demonstrates that social distancing was effective and possible in higher socioeconomic level households.^10^ In addition to increasing transmission risk, the adverse living conditions of BAME communities increase their susceptibility and vulnerability to SARS-COV-2. The COVID-19 pandemic is aggravating racial and societal inequalities; models have demonstrated that overcrowding and population density are important in enabling the continued transmission of infection.^11^

Ethnic minorities comprise a higher proportion of the high-risk and low-paid essential workers in the working-age population, especially in urban areas, thereby increasing their risk of exposure to and acquisition of COVID-19.^7^ In London, 34% of the general working population consists of Black and Asian workers^7^; however, they represent 54%, 48% and 44% of food retail workers, health and social care workers, and transport workers, respectively.^7^ As individuals from these communities are more likely to not have the option of working from home, and may require regular public transport use, they are also exposed to a higher risk of infection by these routes. Furthermore, as these groups have far more social contacts than others, this exposure also puts their work-related contacts and wider households at an increased risk of infection and more severe outcomes.^7^ Lockdown measures have disproportionately affected ethnic minorities that are already experiencing inequalities^12^; unemployment, financial insecurities, housing evictions and mental health issues are experienced at higher levels in Black and Bangladeshi communities when compared to their White counterpart.^12^

## Effective and fair COVID-19 vaccine allocation strategies: targeting ethnic minorities

While the UK passed 100,000 reported COVID-19 deaths in January 2021,^13^ vaccine supplies remain limited.^14^ As countries discuss how to distribute the available vaccines,^11^ developing fair prioritisation strategies and targeted decisions that allocate scarce resources in a way that maximises health benefits is essential.

Direct and indirect benefits are accrued through vaccinations.^15^ Direct protection of individuals from future infection and its negative health outcomes and indirect protection of the population through reduced transmission and risk of infection, including in those who have not been vaccinated, are the key vaccination objectives.^15^

Mitigating the impact of COVID-19 could be achieved through targeted vaccination of all high-risk groups. The World Health Organization and The National Academies of Sciences, Engineering and Medicine both recommend policy-makers allocate COVID-19 vaccines in a manner that prioritises ethnic minorities that are socioeconomically disadvantaged, preventing further exacerbations of existing health inequalities.^16,17^ Securing the greatest benefit across the population will require allocating vaccines to those who have a higher risk of transmission, such as those living in multi-generational households, thereby increasing direct and indirect benefits of the vaccine.^15^ In line with the Centers for Disease Control and Prevention guidance, in preparation for influenza pandemics, vaccines should be allocated in a way that, first, minimises the impact of the pathogen on health and, second, reduces disruption to society and the economy.^18^ In addition to the health and economic impacts of COVID-19 disproportionately affecting ethnic minorities, these communities contribute inordinately to the high-risk key workers who provide critical services – frontline and other – that have allowed the nation to operate as normally as possible and to maintain societal structure during the pandemic. Therefore, prioritising essential workers for vaccination will preserve the healthcare system, accelerate re-opening of society, revive the economy and enable the operation of essential community services. As social justice is the moral foundation of public health,^19^ COVID-19 vaccine allocation should ensure unjust health and socioeconomic inequalities are not further intensified in systematically disadvantaged groups.

Ineffective vaccine allocation strategies likely play a role in the high levels of vaccine hesitancy observed across ethnic minorities.^20^ Studies and surveys suggest that Black communities are most likely to be COVID-19 vaccine-hesitant, followed by Bangladeshi and Pakistani groups.^20^ The UK Government’s Scientific Advisory Group for Emergencies has highlighted the significant risk of low COVID-19 vaccine uptake in ethnic minorities, advising better understanding of the barriers that contribute to low uptake.^20^ Lack of trust in some ethnic minority communities as a result of cultural and structural racism, low confidence in the safety and efficacy of the vaccine, and limited endorsement from trusted providers and community leaders are likely to be key factors^20^; moreover, physical barriers including lack of vaccines, transport access and inconvenience of appointments can also hinder vaccine uptake in these communities.^20^

Effective vaccine allocation strategies can alleviate physical and local barriers faced by ethnic minorities such as vaccine-related access and convenience.^20^ As receiving the vaccine may lead to loss of expenses, resulting from transportation and travel and waiting time, some communities may require practical support with physical barriers to prevent financial losses. Beyond immunisation centres, distributing and administering vaccines in community-based settings that are highly accessible is likely to facilitate and improve uptake,^20^ thereby reducing community spread and the general population’s risk of COVID-19.

## Conclusion

The UK’s vaccine allocation strategies have the potential to further exacerbate the pre-existing, persistent but avoidable, racial inequalities that the COVID-19 pandemic and the wider governmental and societal response have harshly exposed and amplified. Dismissing the racial and socioeconomic disadvantages that ethnic groups face may result in a devastating impact lasting far beyond the end of the pandemic. Controlling further outbreaks and, ultimately, ending the pandemic will require implementation of approaches that target ethnic minorities as well as ensuring that vaccine allocation strategies are effective, fair and justifiable for all.^21^
